# Supplementary material for: Efficacy of different dietary therapy strategies in active pediatric Crohn’s disease: a systematic review and network meta-analysis
Source: PeerJ. 2024 Dec 13;12:e18692. doi: 10.7717/peerj.18692 (PMC11648686; doi:10.7717/peerj.18692)
Supplement: Supplemental Information 2 [file peerj-12-18692-s002.docx]

**Supplementary Material**

**eFigure 1.** Risk of bias graph and summary

**eFigure 2.** Consistency test results of clinical remission rates of different Treatments

**eFigure 3.** Cumulative Ranks and Surface Under the Cumulative Rank Curve (SUCRA) for clinical remission rates

**eFigure 4.** The comparative forest plot for clinical remission rates

**eFigure 5.** Consistency test results of tolerance of different Treatments

**eFigure 6.** Cumulative Ranks and SUCRA for tolerance

**eFigure 7.** The comparative forest plot for tolerance

**eFigure 8.** Cumulative Ranks and SUCRA for c-reactive protein

**eFigure 9.** The comparative forest plot for c-reactive protein

**eFigure 10.** Cumulative Ranks and SUCRA for albumin

**eFigure 11.** The comparative forest plot for albumin

**eFigure 12.** Comparison-adjusted funnel plots

**eFigure 13.** Cumulative Ranks and SUCRA for fecal calprotectin

**eFigure 14.** The comparative forest plot for fecal calprotectin

**eFigure 1.**  Risk of Bias Summary for All Included Studies

**
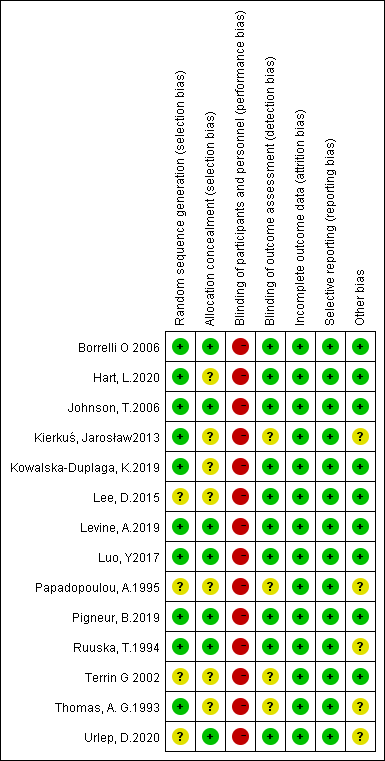
**

**eFigure 2.** Consistency test results of clinical remission rates of different Treatments


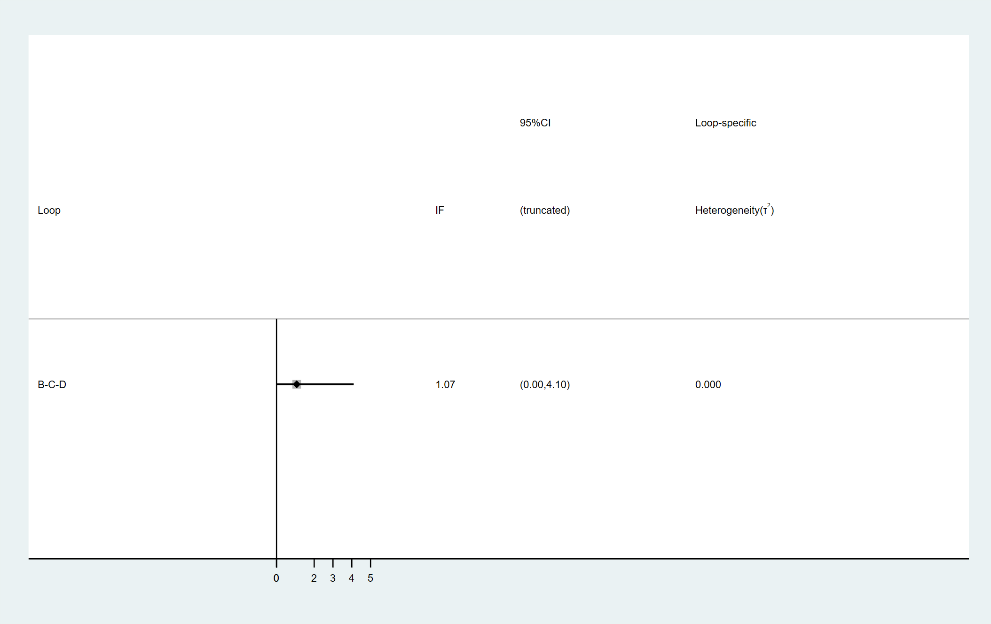


B: exclusive enteral nutrition; C: Infliximab; D: partial enteral nutrition.

**eFigure 3.** the cumulative ranking area for clinical remission rates.


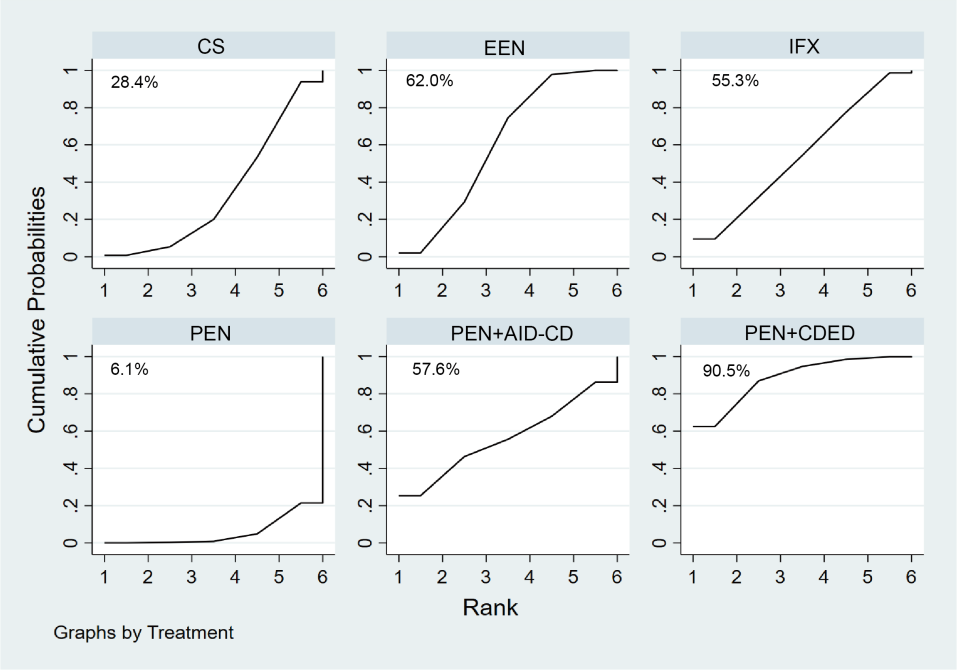


EEN: exclusive enteral nutrition; CS: Corticosteroids; IFX: Infliximab; PEN: partial enteral nutrition; AID-CD: anti-inflammatory diet for Crohn’s disease; CDED: Crohn’s disease exclusion diet.

**eFigure 4.** The comparative forest plot for clinical remission rates

­­­­­­
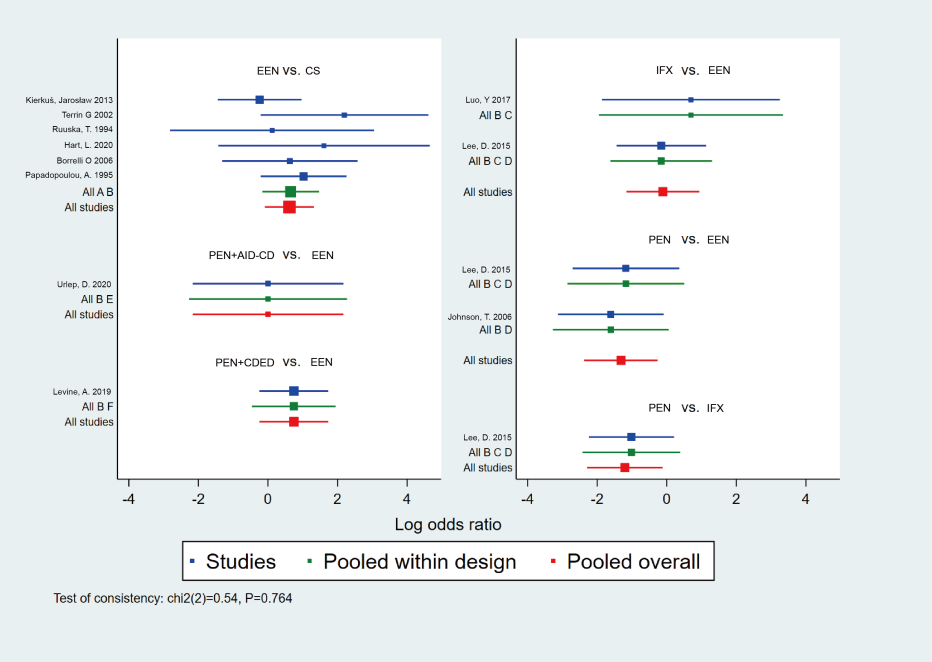


CS: corticosteroids; EEN: exclusive enteral nutrition; IFX: Infliximab; PEN: partial enteral nutrition; AID-CD: anti-inflammatory diet for Crohn’s disease; CDED: Crohn’s disease exclusion diet.

**eFigure 5.** Consistency test results of tolerance of different Treatments

B: exclusive enteral nutrition; C: Infliximab; D: partial enteral nutrition.

**eFigure 6.** Cumulative Ranks and Surface Under the Cumulative Rank Curve (SUCRA) for tolerance
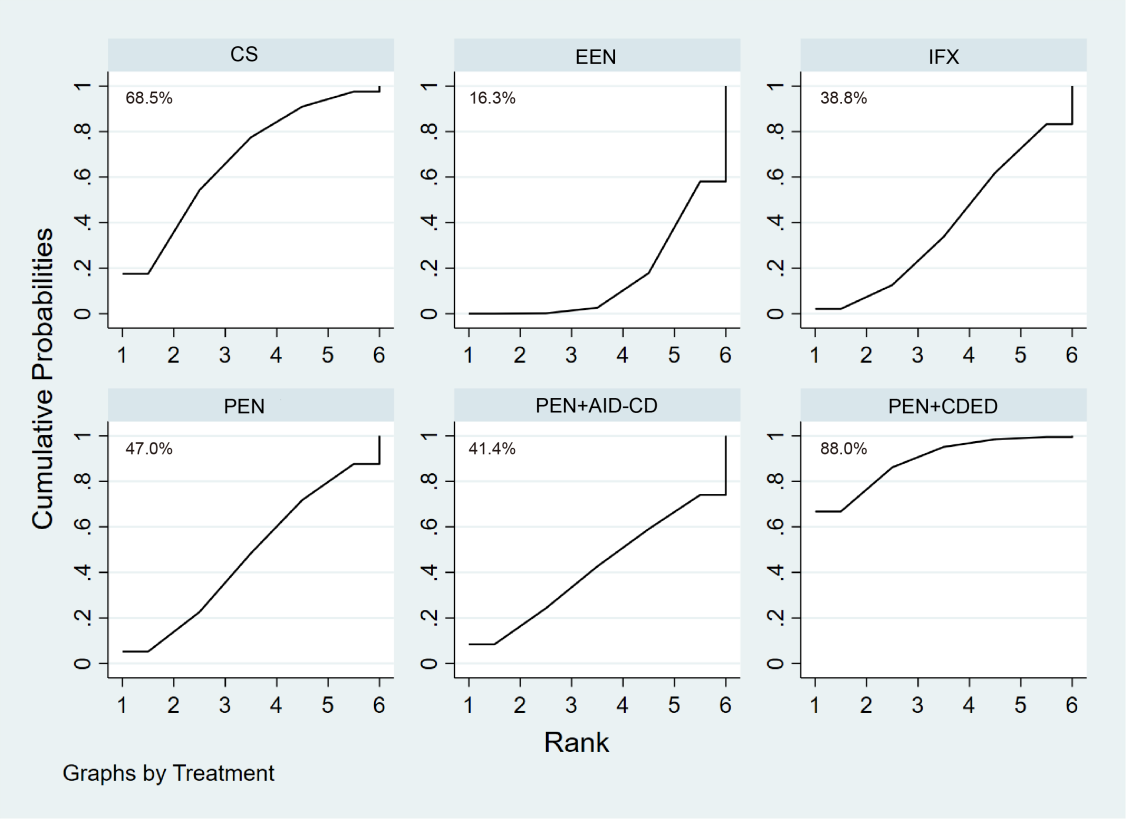


EEN: exclusive enteral nutrition; CS: Corticosteroids; IFX: Infliximab; PEN: partial enteral nutrition; AID-CD: anti-inflammatory diet for Crohn’s disease; CDED: Crohn’s disease exclusion diet.

**eFigure 7.** The comparative forest plot for tolerance


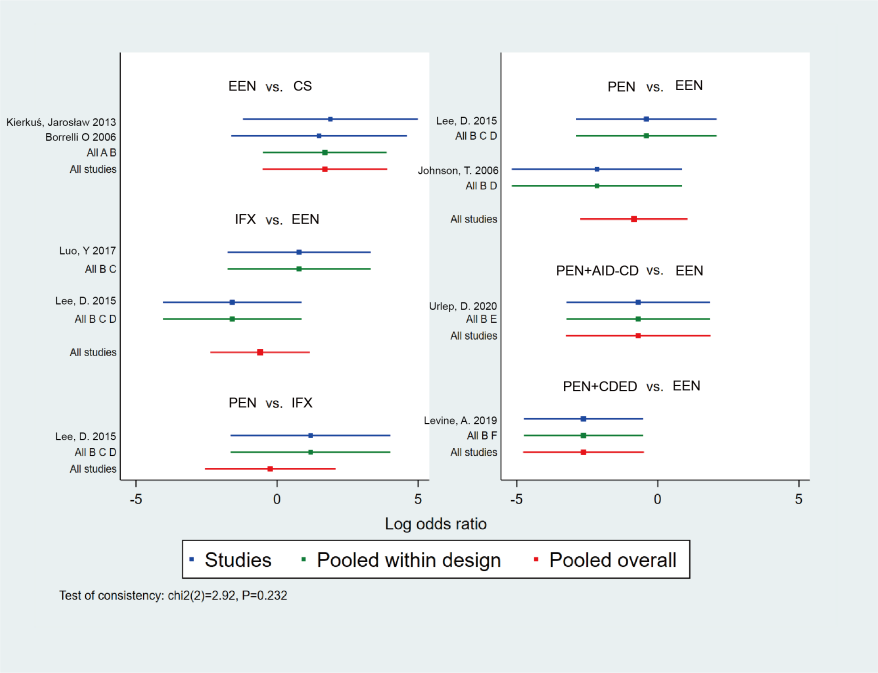


CS: corticosteroids; EEN: exclusive enteral nutrition; IFX: Infliximab; PEN: partial enteral nutrition; AID-CD: anti-inflammatory diet for Crohn’s disease; CDED: Crohn’s disease exclusion diet.

**eFigure 8.** Cumulative Ranks and Surface Under the Cumulative Rank Curve (SUCRA) for c-reactive protein

EEN: exclusive enteral nutrition; CS: Corticosteroids; PEN: partial enteral nutrition; AID-CD: anti-inflammatory diet for Crohn’s disease; CDED: Crohn’s disease exclusion diet.

**eFigure 9.** The comparative forest plot for c-reactive protein

CS: corticosteroids; EEN: exclusive enteral nutrition; PEN: partial enteral nutrition; AID-CD: anti-inflammatory diet for Crohn’s disease; CDED: Crohn’s disease exclusion diet.

**eFigure 10.** Cumulative Ranks and Surface Under the Cumulative Rank Curve (SUCRA) for albumin

EEN: exclusive enteral nutrition; CS: Corticosteroids; IFX: Infliximab; PEN: partial enteral nutrition; AID-CD: anti-inflammatory diet for Crohn’s disease.

**eFigure 11.** The comparative forest plot for albumin

CS: corticosteroids; EEN: exclusive enteral nutrition; IFX: Infliximab; PEN: partial enteral nutrition; AID-CD: anti-inflammatory diet for Crohn’s disease.

**eFigure 12.** Comparison-adjusted funnel plots


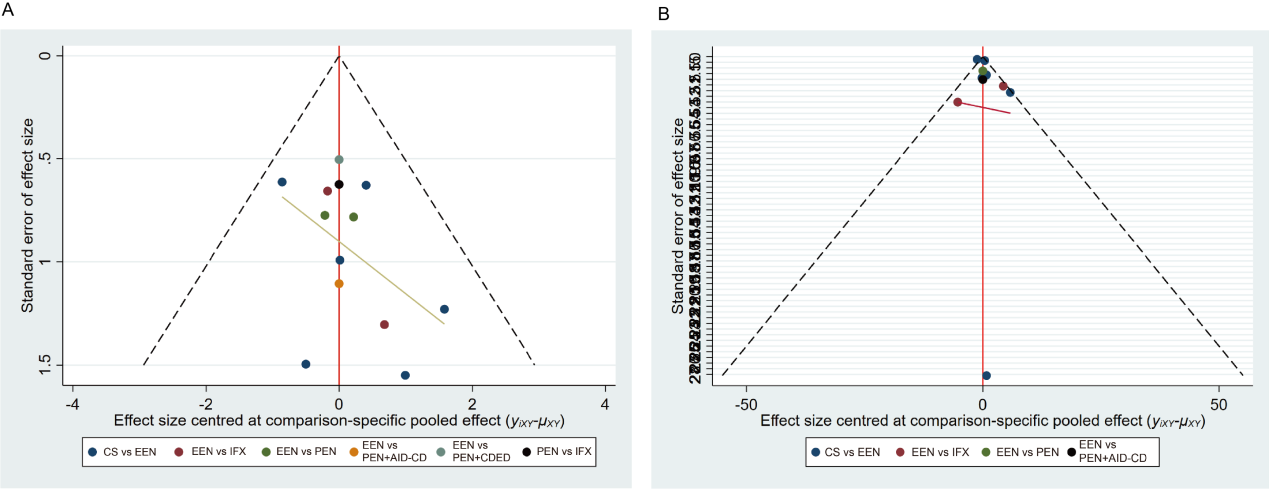
­­

(A) Comparison-adjusted funnel plots of clinical remission. (B) Comparison-adjusted funnel plots of albumin. EEN: exclusive enteral nutrition; CS: Corticosteroids; IFX: Infliximab; PEN: partial enteral nutrition; AID-CD: anti-inflammatory diet for Crohn’s disease; CDED: Crohn’s disease exclusion diet.

**eFigure 13.** Cumulative Ranks and SUCRA for fecal calprotectin


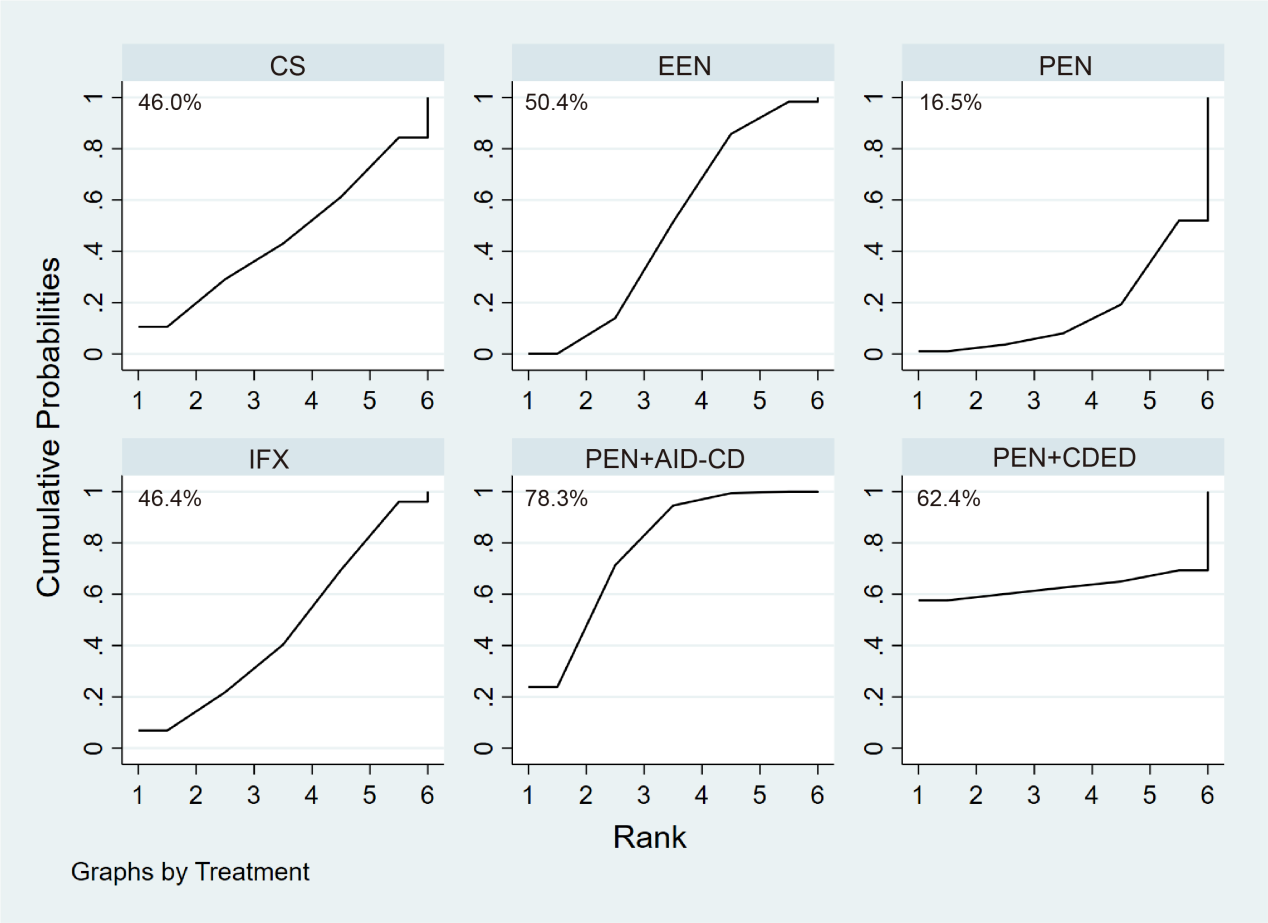
EEN: exclusive enteral nutrition; CS: Corticosteroids; IFX: Infliximab; PEN: partial enteral nutrition; AID-CD: anti-inflammatory diet for Crohn’s disease; CDED: Crohn’s disease exclusion diet.

**eFigure 14.** The comparative forest plot for fecal calprotectin


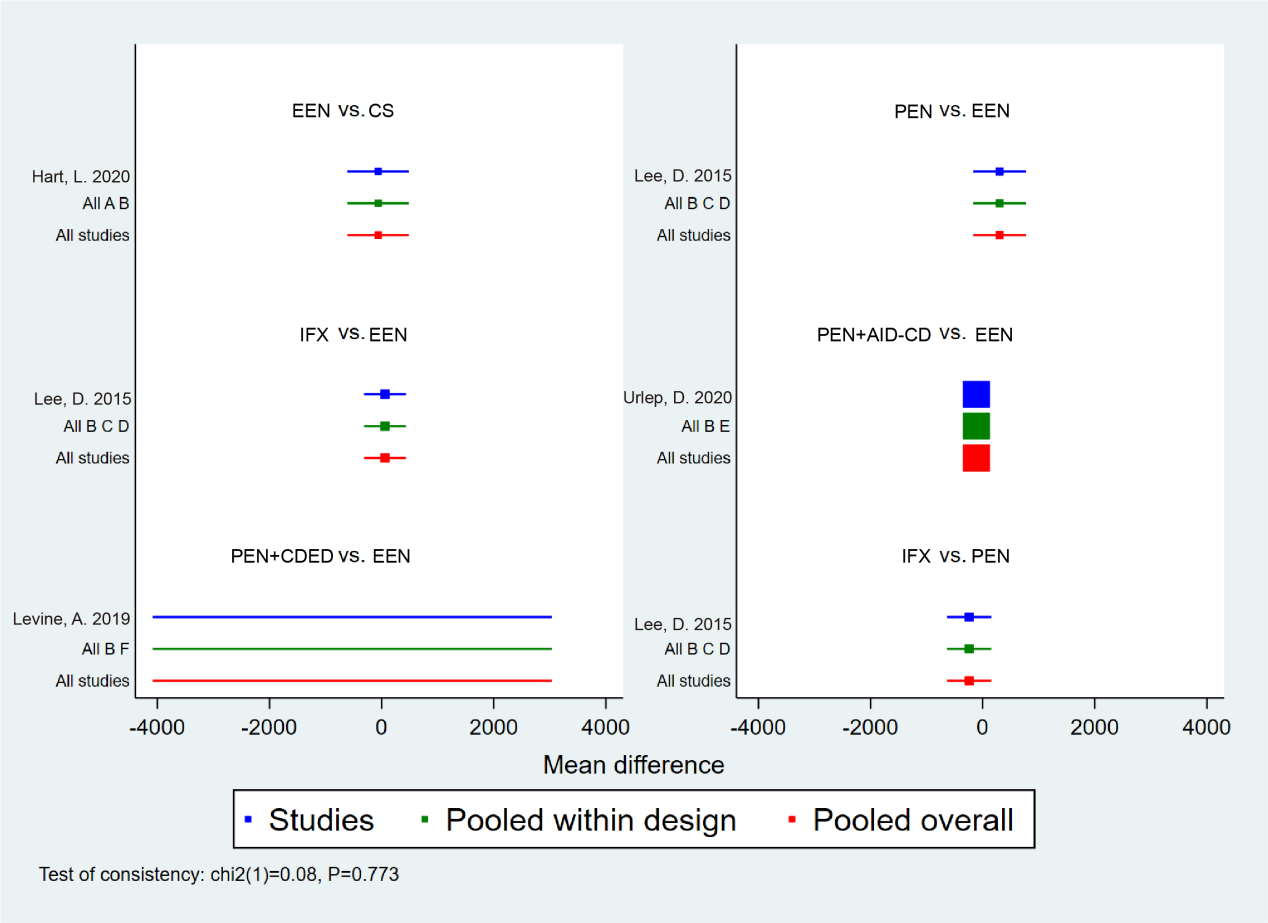


EEN: exclusive enteral nutrition; CS: Corticosteroids; IFX: Infliximab; PEN: partial enteral nutrition; AID-CD: anti-inflammatory diet for Crohn’s disease; CDED: Crohn’s disease exclusion diet.
